# Diagnosis and Treatment of Tuberculosis in the Private Sector, Vietnam

**DOI:** 10.3201/eid1703.101468

**Published:** 2011-03

**Authors:** Nguyen B. Hoa, Frank G.J. Cobelens, Dinh N. Sy, Nguyen V. Nhung, Martien W. Borgdorff, Edine W. Tiemersma

**Affiliations:** Author affiliations: National Tuberculosis Program, Hanoi, Vietnam (N.B. Hoa, D.N. Sy, N.V. Nhung);; Academic Medical Center, Amsterdam, the Netherlands (F.G.J. Cobelens, M.W. Borgdorff, E.W. Tiemersma);; Municipal Health Service, Amsterdam (M.W. Borgdorff);; KNCV Tuberculosis Foundation, The Hague, the Netherlands (E.W. Tiemersma)

**Keywords:** Tuberculosis and other mycobacteria, Vietnam, tuberculosis, private sector, diagnosis, treatment, prevalence, letter

**To the Editor:** In many countries, the private sector (practitioners not employed by government and nongovernment institutions, e.g., hospitals, pharmacies) is a major source of care, even for poor persons, and the area where services for the public are widely available (*1**,**2*). However, little information is available from high-incidence countries about the role of the private sector in tuberculosis (TB) detection and treatment (*3*). In Vietnam, <40% of all TB cases in Ho Chi Minh City (the largest city in Vietnam and with the highest rate of economic growth in the country) were estimated to be treated in the private sector (*4*), and half of all patients with a diagnosis of TB in the public sector (National Tuberculosis Program [NTP]) in Ho Chi Minh City initially sought help in the private sector (*5*). However, this estimate does not reflect private care in the entire country.

In 2006–2007, a countrywide TB prevalence survey was conducted in Vietnam (*6*) in which data were obtained for previous TB treatment. This survey provided an opportunity to calculate a nationally representative estimate of the proportion of TB cases treated in the private sector and to investigate demographic characteristics of persons choosing treatment in this sector.

The study was reviewed and approved by the Research Board of the Vietnam National Lung Hospital. Details of survey methods have been reported (*6*). All eligible persons were screened to identify suspected cases of TB by using a short, structured, screening questionnaire and chest radiograph. Persons with suspected TB were those who reported persistent productive cough, who had radiographic abnormalities suggestive of TB, or who received TB treatment either currently or in the 2 years preceding the survey. Persons had an in-depth interview that included questions on where they were treated for TB. Assessment of socioeconomic status was based on 9 household characteristics (*7*).

Missing data were imputed by using multiple imputation methods, assuming that these data were missing at random to adjust for nonparticipation and missing data on facility of TB treatment (*8*). We used the ice and mi commands in Stata version 11 software (StataCorp LP, College Station, TX, USA), which included age, area, zone, and socioeconomic status.

Of the 103,924 eligible persons in selected districts, 94,179 (91%) were screened, 7,498 were identified as having suspected TB, and 407 reported having been recently treated for TB: 316 (77.6%) in public health facilities (PHFs) reporting cases to the NTP, 8 (2.0%) in PHFs not reporting cases to the NTP, and 29 (7.1%) in private health care facilities not reporting to the NTP. Fifty-four (13.3%) did not provide information about where they were treated. Multiple imputation led to adjusted proportions of 88.9%, 2.9%, and 8.2%, respectively. Sensitivity analyses, which assigned 54 persons with missing data for location of TB treatment to PHFs or private clinics, resulted in a range of 7.1%–20.3% for private sector treatment.

Characteristics of participants by type of facility where they received TB treatment are shown in the Table. Women, younger persons, and residents of southern Vietnam were more likely to seek treatment in the private sector. Urban populations and those with the highest socioeconomic status were most likely to seek private care, but these differences were not significant (Table).

**Table:** Characteristics of patients treated for tuberculosis at time of prevalence survey (2006–2007) or in 2 preceding years, Vietnam*

Characteristic	Public health facilities reporting to NTP, no. (%)	Public health facilities not reporting to NTP, no. (%)	Private sector, no. (%)	OR (95% CI)
Total	316 (89.5)	8 (2.3)	29 (8.2)	NA†
Sex				
M	230 (92.4)	4 (1.6)	15 (6.0)	1
F	86 (82.7)	4 (3.8)	14 (13.5)	2.4‡ (1.0–5.6)
Age, y				
15–35	51 (79.7)	2 (3.1)	11 (17.2)	3.1 (1.3–7.4)
35–55	134 (93.1)	4 (2.8)	6 (4.2)	0.4 (0.1–0.9)
>55	131 (90.3)	2 (1.4)	12 (8.3)	1.0 (0.4–2.3)
Area				
Urban	95 (85.6)	2 (1.8)	14 (12.6)	2.2 (0.9–5.1)
Remote	59 (89.4)	2 (3.0)	5 (7.6)	0.9 (0.3–2.5)
Rural	162 (92.0)	4 (2.3)	10 (5.7)	0.5 (0.2–1.2)
Zone				
Northern	117 (93.6)	5 (4.0)	3 (2.4)	0.2 (0.0–0.7)
Central	50 (87.7)	1 (1.8)	6 (10.5)	1.4 (0.4–3.8)
Southern	149 (87.1)	2 (1.2)	20 (11.7)	2.5 (1.1–6.5)
Socioeconomic status§				
Lowest	104 (91.2)	2 (1.8)	8 (7.0)	0.8 (0.3–1.9)
Medium	85 (90.4)	2 (2.1)	7 (7.4)	0.9 (0.3–2.2)
Highest	103 (88.0)	2 (1.7)	12 (10.3)	1.5 (0.6–3.5)
No information	24 (85.7)	2 (7.1)	2 (7.1)	NA¶

*NTP, National Tuberculosis Program; OR, odds ratio, CI, confidence interval; NA, not available. †Not available because >4 categories are needed for calculation of OR. ‡For difference between private and public sectors. All other comparisons were for differences between 1 group and the other groups combined. §Based on a set of indicators (*7*) and expressed by tertiles of expenditure distribution among all survey participants. ¶Not available because there were only 28 persons with no information.

We estimated that 8.2% of persons with TB in Vietnam were treated in private clinics. Although sensitivity analysis showed a wide range around this estimate (7.1%–20.3%), our data suggest that private health care facilities treat a large proportion of TB patients. Use of the private sector was relatively high in southern Vietnam (11.7%), especially in southern urban areas (13.3%).

Taking into account observed patterns in our study (i.e., preference for private sector treatment among young persons, higher income groups, and those in urban areas) and general development in Vietnam, we expect that the private sector will provide increased diagnosis and treatment of TB. With availability of TB drugs in private pharmacies (*9*), improved private TB care is needed (*10*) by establishing better collaboration and coordination between the NTP and the private sector through the public–private approach (*4*) and by improving and expanding reporting systems so that all facilities where TB patients are diagnosed and treated are included.

A strength of our study is that it included a nationally representative sample of previously treated TB patients because all who reported having been treated recently for TB were defined as persons with suspected TB. A limitation of our study is that it depended on self-reported TB treatment, which is prone to recall bias and has potentially led to underestimation because some participants may not have been accurate regarding previous or current episodes of TB and private sector treatment because of social desirability bias. The NTP in Vietnam needs to implement and improve public–private mix projects, and private practitioners need to be appropriately trained to report TB patients according to NTP guidelines.
